# Supplementary material for: Identification of photoperiod-induced specific miRNAs in the adrenal glands of Sunite sheep (Ovis aries)
Source: Front Vet Sci. 2022 Jul 22;9:888207. doi: 10.3389/fvets.2022.888207 (PMC9354845; doi:10.3389/fvets.2022.888207)
Supplement: Supplementary file 1 [file Data_Sheet_1.ZIP › Supplementary file/miRDeep2 file.zip!/miRDeep2 file/Novel_Structure/SPLP42A3/Novel_705.pdf]

Provisional ID	:	NC_019484.2_38339
Score total	:	18.8
Score for star read(s)	:	3.9
Score for read counts	:	11.3
Score for mfe	:	2
Score for randfold	:	1.6
Score for cons. seed	:	
Total read count	:	34
Mature read count	:	22
Loop read count	:	0
Star read count	:	12

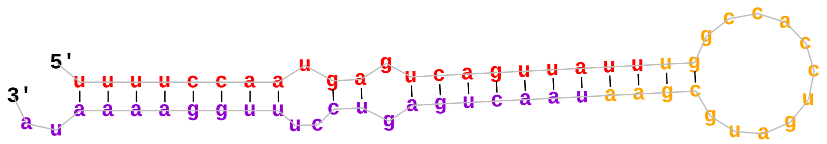

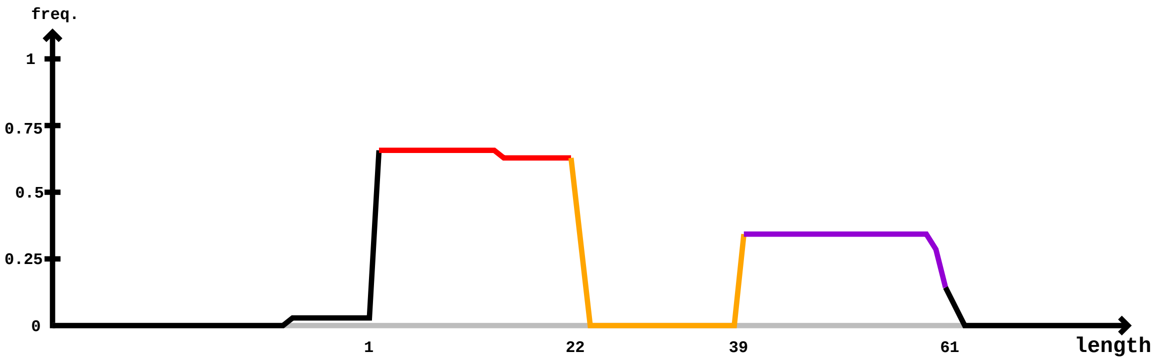


```
                                   Mature                   Star
5'- ucucccuguuuuaaaacuucaacaucagggucuuuuccaaugagucaguuauuuggccaccugaugcgaauaacugaguccuuuggaaaauacccugaugcugggaaaga  -3'   obs
    ucucccuguuuuaaaacuucaacaucagggucuuuuccaaugagucaguuauuuggccaccugaugcgaauaacugaguccuuuggaaaauacccugaugcugggaaaga        exp
    ..((((.(((....))).....(((((((((.((((((((.((.(((((((((((...........))))))))))).))..)))))))).)))))))))..))))....   reads    mm     sample
    .......................aucagggucuuuuccaaugagA.................................................................   1        1      seq
    ................................uuuuccaaugagucaguuauu.........................................................   17       0      seq
    ................................uuuuAcaaugagucaguuauu.........................................................   1        1      seq
    ................................uuuuccaaugGgucaguuauu.........................................................   1        1      seq
    ................................uuGuccaaugagucaguuauu.........................................................   1        1      seq
    ................................uuuuccaaugagCcaguuauu.........................................................   1        1      seq
    ................................uuuuccaaugagucaguuauuA........................................................   1        1      seq
    ......................................................................uaacugaguccuuuggaaaa....................   2        0      seq
    ......................................................................uaacugaguccuuuggaaaau...................   5        0      seq
    ......................................................................uaacugaguccuuuggaaaaua..................   5        0      seq
```
